# The modular cardiac rhythm management system: the EMPOWER leadless pacemaker and the EMBLEM subcutaneous ICD

**DOI:** 10.1007/s00399-018-0602-y

**Published:** 2018-10-31

**Authors:** F. V. Y. Tjong, B. E. Koop

**Affiliations:** 1 AMC Heart Center, Department of Clinical and Experimental Cardiology, Amsterdam University Medical Center, Location Academic Medical Center, University of Amsterdam, Amsterdam, The Netherlands; 2 Boston Scientific Corporation, St. Paul, MN USA

**Keywords:** Tachycardia, Leadless pacing, Antitachycardia pacing, Subcutaneous implantable cardioverter–defibrillator, Arrhythmias, cardiac, Tachykardie, Sondenlose Schrittmacher, Antitachykarde Schrittmacher, Subkutaner implantierbarer Kardioverter-Defibrillator, Kardiale Arrhythmien

## Abstract

Cardiac implantable electronic devices have been successfully treating patients with brady- and tachyarrhythmias for decades. However, there are still significant complications related to this therapy modality, many related to the transvenous lead. Paradigm-shifting technologies, such as the subcutaneous implantable cardioverter–defibrillator (S-ICD) and leadless cardiac pacemakers (LCP), have emerged to address these complications. The novel modular cardiac rhythm management (mCRM) system, consisting of a communicating antitachycardia pacing-enabled LCP and S‑ICD, is the first system to integrate wireless intrabody communication between devices to allow for coordination of leadless pacing and defibrillator therapy delivery. In this review, the design and concept of the mCRM system are presented and available evidence is summarized.

## Zusammenfassung

Seit Jahrzehnten werden kardiale implantierbare elektronische Devices erfolgreich bei Patienten mit Brady- und Tachyarrhythmien eingesetzt. Bei dieser Therapie gibt es jedoch noch immer bedeutende Komplikationen, von denen viele auf die transvenöse Elektrode zurückzuführen sind. Ein Paradigmenwechsel entstand durch Technologien wie den subkutanen implantierbaren Kardioverter-Defibrillator (S-ICD) sowie sondenlose Herzschrittmacher (LCP), die entwickelt wurden, um diese Komplikationen zu adressieren. Das neue modulare Herz-Rhythmus-Management(mCRM)-System, bestehend aus einem kommunizierenden LCP mit antitachykarder Stimulation und einem S‑ICD, ist das erste System, welches die kabellose Kommunikation zwischen den Devices innerhalb des Körpers integriert, um die Koordination der sondenlosen Schrittmacher- und Defibrillatortherapie zu ermöglichen. In diesem Review werden Design und Konzept des mCRM-Systems vorgestellt sowie die verfügbare Evidenz zusammengefasst.

## Introduction

**Fig. 1:**
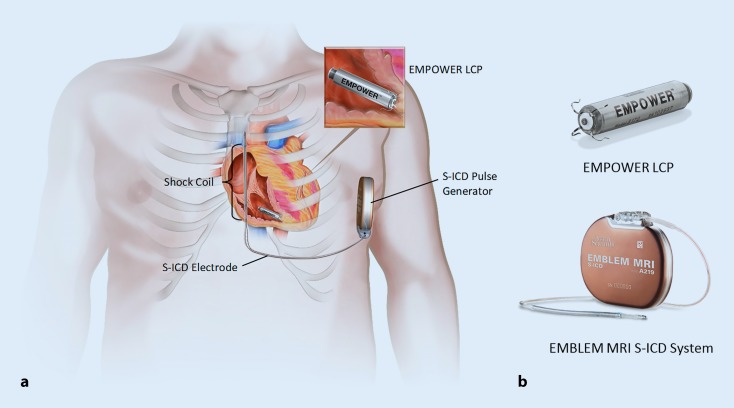
The modular cardiac rhythm management (mCRM) system.** a** Depiction of implanted mCRM system. **b** Close-up images of EMPOWER and EMBLEM MRI devices (not to scale). *S-ICD* subcutaneous implantable cardioverter–defibrillator, *LCP* leadless cardiac pacemaker. © 2017 Boston Scientific Corporation or its affiliates. All rights reserved. Used with permission of Boston Scientific Corporation

For many decades, cardiac electrical implantable devices (CIED) have been the cornerstone therapy for patients with brady- and tachyarrhythmias [1–4]. Despite many years of innovation and device development, these systems are still associated with a significant amount of acute and chronic complications, of which many are related to the “weakest link” of these systems: the transvenous lead [5–7]. Paradigm shifting technologies have been introduced to eliminate the need for a transvenous lead. First, in 2008, the subcutaneous implantable cardioverter defibrillator (S-ICD) was introduced [8], a device placed fully subcutaneously, limiting intracardiac hardware. Improvements in battery technology and advanced electrical circuitry brought about leadless pacemakers, miniaturized self-contained intracardiac single-chamber pacemakers delivered in the right ventricle through a percutaneous transfemoral catheter based approach [9, 10]. Two systems have been clinically available now for several years and showed clinical efficacy and safety [9–13]. However, existing leadless pacing systems and the S‑ICD are only available for patients requiring single-chamber right ventricular (RV) pacing or shock-only defibrillation therapy, respectively. Combined use of both types of devices could bring the potential benefits of this leadless approach to a larger patient population. Adding antitachycardia pacing (ATP) to this combined system would enable even larger patient groups to potentially benefit [14]. These combined device systems require safe and reliable device–device communication and enable a novel treatment concept of modular cardiac rhythm management (mCRM) therapy, in which device therapy could be further personalized to the current and future patients’ needs. The first mCRM system is presented in this review and consists of a communicating and the EMPOWER™ leadless cardiac pacemaker (LCP; both Boston Scientific), which are shown in Fig. 1. To date, this is the first system consisting of communicating independent cardiac devices for synchronized therapy application.

## Intrabody communication and the modular cardiac rhythm management system

Beginning in the 1990s, researchers became interested in the possibilities of intrabody communication involving implanted devices in various forms and using various communication modalities [15–17]. It later became clear that one of the most energy efficient modalities for communication between implanted devices was galvanic coupling, or communication using the conductive properties of the body [18–20]. Through galvanic coupling, one implanted device transmits low-energy electrical signals, such as via alternating current, which is detected by two electrodes on the receiving device. The advantages of galvanic coupling over other communication modalities such as radiofrequency (RF) communication include not only lower energy requirements but also lower signal losses in tissue. The disadvantages of galvanic coupling include comparatively lower rates of data transmission and the potential for larger null spaces due to relative physical orientation of device electrodes.

Due to the power efficiency and less lossy nature in human tissue, galvanic coupling, or “conducted” communication, was selected as the communication modality to be used for device–device communication in the mCRM system. A secondary design goal was to allow existing implanted S‑ICDs to be upgraded via firmware, with no change to the hardware within the system, to enable the ability to communicate. The advantage of this strategy is to allow access to this new technology, the modular addition of intracardiac therapy, for patients with an EMBLEM platform S‑ICD system without the need to explant and replace the S‑ICD pulse generator. The compromise is to utilize only unidirectional conducted communication from S‑ICD to LCP, which allows access to ATP therapy but limits additional features that could come from this pairing to future generations of S‑ICD pulse generator where bidirectional communication would be possible.

**Fig. 2:**
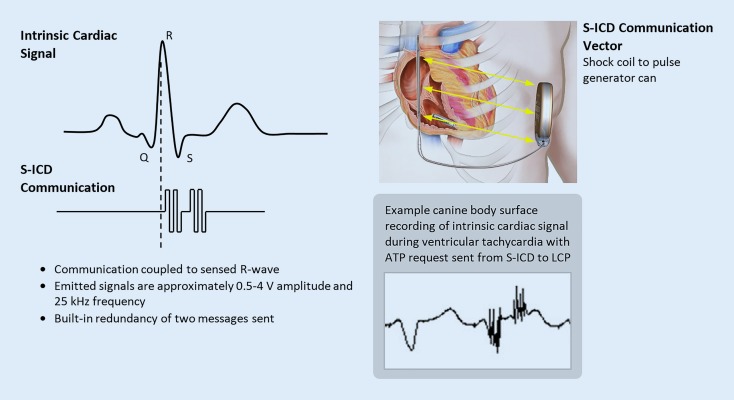
Device–device communication of the mCRM system. Depiction of how communication messages are sent from S‑ICD to EMPOWER LCP via conducted signals. The EMPOWER LCP senses the conducted signals via its cathode and anode, which are also used for sensing intrinsic cardiac signals. *mCRM* modular cardiac rhythm management, *S-ICD* subcutaneous implantable cardioverter–defibrillator, *LCP* leadless cardiac pacemaker, *ATP* antitachycardia pacing

To accomplish the objective of adding conducted communication ability to the S‑ICD system without updating its hardware, existing circuitry in the S‑ICD pulse generator, normally utilized to check shock circuit integrity, was repurposed to send bursts of approximately 25 kHz pulses from the shock coil of the S‑ICD electrode to the S‑ICD pulse generator can. These pulses are low in voltage amplitude (<5 V emitted) and are sent in a proprietary pattern that can be recognized by the LCP and is distinct from various levels of signal noise that may be sensed by the LCP. The pattern of pulses constituting a full communication message from S‑ICD requires less than 40 ms in duration, though to provide redundancy two messages are sent in series to help mitigate risk of failed receipt by the LCP. Two full communication messages back-to-back require less than 135 ms in duration, which is short enough to fit within a standard pacemaker cardiac sensing refractory window. S‑ICD communication messages are sent coincident with the R‑wave of the cardiac cycle to avoid oversensing of these communications on the LCP’s cardiac sensing channel. Fig. 2 depicts the S‑ICD-to-LCP communication details.

Since the vector used by the S‑ICD to communicate electrical signals is from the shock coil to the pulse generator can, and the LCP senses these conducted signals via its cathode and anode, the spatial orientation of the LCP within the S‑ICD communication vector can impact the ability of the LCP to sense the transmitted signals from S‑ICD. Theoretically, the highest communication signal amplitude sensed by the LCP is when its main axis is aligned with the S‑ICD communication vector as shown in Fig. 2. Conversely, the lowest communication signal amplitude sensed by the LCP is when its main axis is perpendicular to the S‑ICD communication vector, or when the LCP is approximately parallel with the S‑ICD shock coil when the implanted system is viewed in an anterior–posterior view under fluoroscopy. The orientation of the LCP relative to the S‑ICD communication vector in humans is expected to have a favorable position in relation to the communication vector, with a preferred device deployment in the right ventricular apicoseptal region. Implantation of the LCP in the free wall of the right ventricle is not recommended because of the less favorable device orientation and the higher potential risk of cardiac perforation. A previous report evaluated the device orientation of other leadless pacemakers in humans (e. g., [21]). However, if the EMPOWER LCP were to be in an unfavorable orientation, the combination of S‑ICD communication message redundancy and the motion of the heart during the cardiac cycle is intended to mitigate the possibility of the S‑ICD communication not being received by the LCP.

In terms of tachyarrhythmia and bradyarrhythmia sensing and therapy, the S‑ICD and LCP elements of the mCRM system act independently from each other except for antitachycardia pacing (ATP) therapy. For ATP, S‑ICD employs the same sensing and detection criteria to declare a tachyarrhythmia episode as those used in market-approved A209 and A219 model S‑ICDs. However, the same model S‑ICD with firmware upgrade and mCRM communication feature activated can request the EMPOWER LCP to perform ATP therapy according to programmed parameters. These programmable parameters include the number of ATP attempts within a programmed S‑ICD tachyarrhythmia rate zone, scheme of ATP (Burst or Scan), ATP coupling/burst interval, and number of pulses per ATP attempt. Depending on the rate zone of the S‑ICD in which tachyarrhythmia is occurring, ATP therapy can be requested of the LCP, including multiple times, before moving to charging for shock therapy, or ATP therapy can be requested and applied in parallel to charging for shock. After the ATP communication request by the S‑ICD there is a programmed delay of about 4 s before the S‑ICD starts sensing the intrinsic rhythm to allow ATP therapy to be applied by the LCP and ensure ATP is not included in the subsequent tachydetection by the S‑ICD.

## Data from acute and chronic animal implants

Data regarding successful S‑ICD/LCP device–device communication and coordination of ATP during simulated ventricular tachycardia in an ovine model was first reported in 2016 [22]. A larger animal study was published later with more extensive evaluations of the technology [23]. In the latter study, acute and chronic animal implants of the mCRM system were completed to test the pacing and sensing performance of the EMPOWER LCP, as well as the communication and system performance of the S‑ICD and LCP together. Data from acute implants in multiple animal species (canine, ovine, and swine) and up to 90 days of chronic implant in canines were reported, with LCP pacing and sensing data as shown in Table 1.

**Table 1:** Acute and 3‑month EMPOWER LCP performance showing favorable pacing and sensing performance over the course of the study. Values are *n* (%) or mean ± standard deviation

	Acute Performance (*n* = 40)			3-Month Performance (*n* = 23)			
	Baseline			Canine			
	Ovine (*n* = 8)	Swine (*n* = 5)	Canine (*n* = 27)	7 Days	28 Days	90 Days	*p* Value
Implant Success	8 (100)	5 (100)	26 (96)	–	–	–	–
**LCP Position**							
RV Apex	8 (100)	4(80)	12 (46)	–	–	–	–
RV Apical Septum	0	0	14 (54)	–	–	–	–
RV Outflow Tract	0	1 (20)	0	–	–	–	–
**LCP Electrical Performance**	**(*n*** **= 8)**	**(*n*** **= 5)**	**(*n*** **= 26)**	**(*n*** **= 23)**	**(*n*** **= 23)**	**(*n*** **= 23)**	
Pacing Threshold, V at 0.5 ms	1.10 ± 0.81	0.53 ± 0.49	0.37 ± 0.19	0.56 ± 0.37^b^	0.54 ± 0.30^b^	0.72 ± 0.45^b,c^	<0.001
R-Wave Amplitude, mV	6.6 ± 1.4	28.3 ± 5.8	25.8 ± 5.1^d^	26.3 ± 6.8^d^	25.0 ± 9.4^d^	23.3 ± 9.4	<0.001
Impedance, Ω	665 ± 225	753 ± 118	826 ± 171^d^	785 ± 129^d^	827 ± 105^d^	728 ± 141^c^	0.04
**LCP Post Shock Performance**	**(*n*** **= 8)**	**(*n*** **= 2)**	**(*n*** **= 1)**			**(*n*** **= 7)**	
Pre- to Post Shock Change in Pacing Threshold, V at 0.5 ms	0.0 ± 0.5	0.1 ± 0.1	0	–	–	0.0 ± 0.1	–
Pre- to Post-Shock Change in Impedance, W	18 ± 49	26 ± 40	5	–	–	−48 ± 58	–

Table reprinted from Tjong et al. [23] with permission from the publisher

*LCP* leadless cardiac pacemaker, *RV* right ventricle

^a^*p* value between baseline and 90 days, for pacing threshold and impedance tested with the Student t test, for R wave calculated with a linear regression analysis

^b^Pacing threshold data from 3 animals were excluded because the LCP prototypes did not have steroid-eluting electrode

^c^Impedance and pacing threshold data from 1 animal were excluded due to suspected device malfunction at day 90

^d^R-wave and impedance data from 7 animals at baseline, 9 animals at day 7, and 7 animals at day 28 were excluded due to programmer software malfunction

**Fig. 3:**
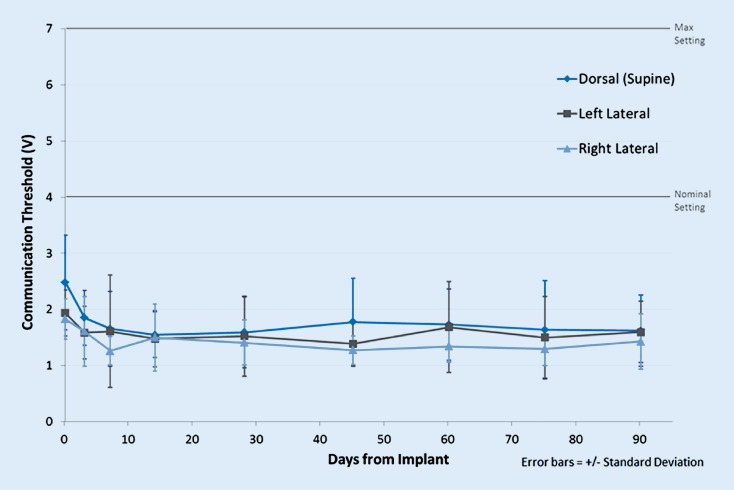
Device–device communication thresholds. Threshold for successful S‑ICD to LCP communication, where the threshold is based on the voltage amplitude setting in the S‑ICD used for sending the communication pulses. This is not equal to the voltage emitted by the S‑ICD, which is generally 30–75% lower. The communication voltage amplitude setting can range from 1 to 7 V, with a nominal setting of 4 V. *S-ICD* subcutaneous implantable cardioverter–defibrillator, *LCP* leadless cardiac pacemaker. Reprinted from Tjong et al. [23] with permission from the publisher

Data obtained on voltage threshold for successful communication from S‑ICD to LCP was also obtained in canines for three postures in the same 3‑month study and is shown in Fig. 3. The data were taken with respect to the voltage amplitude setting in the S‑ICD pulse generator used to generate the communication pulses. This setting is not equal to the voltage emitted by the device, which is approximately 30–75% lower than the voltage amplitude setting (depending on the setting used).

These results are important for understanding the chronic stability of device–device communication for the mCRM system in vivo. Specifically, the results show that, on average, the communication threshold was low (signal margin for successful device–device communication was high) and stable throughout the 3‑month study. Further, any differences in communication threshold between the three postures tested were small and not of practical significance relative to the signal margin that exists if the S‑ICD was programmed to its nominal amplitude setting. Indeed, there is little incentive to program the system to a communication setting lower than the nominal value as there is expected to be very low impact to S‑ICD longevity (days or weeks depending on the number of ambulatory ATP episodes) to send communications during the device lifetime, regardless of amplitude setting.

**Fig. 4:**
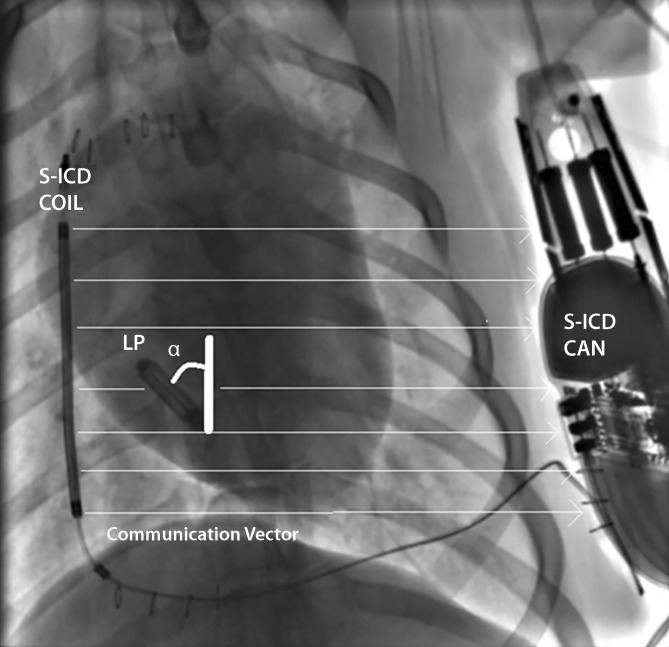
Device orientation in communication vector between S‑ICD and LCP. This fluoroscopy image shows the measurements of the orientation vector between S‑ICD lead and can, with the orientation of the LCP within this vector (angle α). *S-ICD* subcutaneous implantable cardioverter–defibrillator, *LP* leadless pacemaker. Reprinted from Tjong et al. [23] with permission from the publisher

Using data from the same 3‑month animal study, an analysis of the impact of EMPOWER LCP spatial orientation on communication threshold was conducted and reported in 2018 [24]. Included in the same evaluation was a retrospective analysis of patients from a single center implanted with either an S‑ICD system or a commercially available leadless pacemaker. This analysis was performed to utilize the data from the animal study to determine the favorability of the LCP spatial orientation relative to the S‑ICD communication vector in human anatomy. An example of the communication vector between S‑ICD and LCP and the orientation of the LCP within this vector is shown in Fig. 4. The findings from the animal data showed that a when the LCP is approximately parallel with the S‑ICD shock coil in an anterior–posterior view under fluoroscopy, the communication threshold trends higher but the effect was moderate. It is hypothesized that the practice of sending two communication messages in series from the S‑ICD helps alleviate the possibility of communication failure. Additionally, the human body is not a homogenous conductive medium; organs such as the lungs likely cause a divergence of electric field orientation created by the S‑ICD communication pulses which may allow a high tolerance for many LCP spatial orientations S‑ICD relative to the communication vector. Overall, the angle (α, Fig. 4) of the LCP axis relative to the S‑ICD shock coil in canines was found to be 25° on average, while in humans the mean angle was 56°. This result may predict an even higher signal margin for communication when the mCRM system is evaluated in humans in the near future, though it is acknowledged there are other important factors such as torso size which can impact communication success.

## Discussion

Modular therapy is the idea that one can tailor implantable medical device therapy to the specific needs of the patient throughout their lifetime so that the patient receives only the device(s) they need, only when needed. Medical devices inherently involve risks to the patient related to implantation and to continuing operation in the body. Ideally for the patient the benefits of the implanted devices far outweigh the risks. However, some patients who would be eligible to receive an implanted S‑ICD system instead receive a transvenous ICD system despite having no immediate need for pacing therapy or ATP. This may be a decision made by the physician with a view to the possibility the patient would need these therapies in the future. The downside of this decision is the patient receives a system that has disadvantages compared to S‑ICD: Intracardiac leads which may fail, infection pathways to the heart, and complications that occur acutely or chronically due to implantation within the heart.

With the advent of the mCRM system and wireless communication capabilities of implants, there will be an alternative approach: Implant the S‑ICD system in eligible patients now and implant the EMPOWER LCP later only if the patient develops a therapeutic need for the device. The number of patients who develop such a need are expected to be low [25], meaning more patients can benefit from a less invasive device therapy strategy. As further components of this modular system are developed in the future, further therapies will be available for application in a modular fashion as the patient’s disease state progresses, heralding an era of truly personalized device medicine.

## Limitations and next steps

The main limitations in understanding the safety and effectiveness of the mCRM system is that only animal data exist currently. Though animal evaluations in multiple models can serve as a useful surrogate when human evaluation is not available, there remain aspects of any animal model which cannot fully replicate human anatomy and environment. Human clinical studies of the EMPOWER LCP as a standalone device and the mCRM system in humans are expected to commence in 2019. Future iterations of the mCRM system are expected to include leadless devices for pacing other chambers of the heart to provide dual chamber pacing therapy or cardiac resynchronization therapy, as standalone devices and in coordination with a co-implanted S‑ICD. Future iterations of S‑ICD system are also expected to allow bidirectional communication with other leadless devices in the mCRM system, as well as new algorithms to allow sensing verification, new discrimination capabilities and new features via device–device communication.

## Practical conclusion

- Complications with the transvenous lead have been overcome using S‑ICD and LCP.
- A modular cardiac rhythm management (mCRM) system, consisting of a communicating antitachycardia pacing-enabled EMPOWER™ LCP and EMBLEM™ platform S‑ICD system, allows for coordination of leadless pacing and defibrillator therapy delivery.
- Firmware can be upgraded in implanted S‑ICDs without explantation or replacement of the pulse generator.
- Acute and 3‑month data regarding device–device communication and coordination of ATP during simulated ventricular tachycardia in animal studies are promising.
- Advances include the possibility to implant the S‑ICD system in eligible patients now and implant the EMPOWER LCP later only if the patient develops a therapeutic need for the device.
- Human clinical studies with the EMPOWER LCP and the mCRM system are expected in 2019.
